# The patients’ perspective of bedside teaching in the post-Covid era

**DOI:** 10.1007/s11845-025-03900-z

**Published:** 2025-02-08

**Authors:** Angela Kearns, James Lennon, Lucy Hurley

**Affiliations:** 1 University of Galway School of Medicine, Mayo Medical Academy, Castlebar, County Mayo Ireland; 2 University of Galway, Newcastle Road, Galway, Ireland

**Keywords:** Bedside teaching, Covid, Medical students

## Abstract

**Background:**

Bedside teaching with real patients is vital to the education of medical students. With the onset of the Covid-19 pandemic in 2020, all student-patient interactions were suspended in order to prevent the spread of the virus. Research has previously focussed on the medical professional and medical student views concerning bedside teaching, with less of a focus on patient insights.

**Aims:**

The purpose and aims of this study were to explore the patient’s perspectives of encountering medical students and contributing to their learning in the clinical setting, beyond the Covid restrictions.

**Methods:**

Qualitative semi-structured interviews were carried out with a cohort of Mayo University hospital inpatients, who had participated in a bedside teaching session. An interview guide was employed to facilitate consistent and analogous data. An interpretative phenomenological approach was used for data analysis yielding a number of themes.

**Results:**

Participants recognised the value of their involvement in the clinical education of future doctors, in addition to the positive impacts of such contributions for their own future healthcare. Participants had no concerns about partaking in bedside teaching sessions relating to Covid infection.

**Conclusions:**

This study assessed the opinions of hospital inpatients with regards to participating in clinical teaching sessions and found that they do not perceive their role as passive.

## Introduction

Bedside teaching has traditionally been a key element in the undergraduate curriculum for medical students. Countless studies have shown its benefits from promoting active learning whilst building clinical skills, to enhancing the development of key humanistic behaviours through role modelling [1–3]. Research suggests that there are four factors that affect bedside teaching: curriculum, clinical tutors, medical students and patients [4]. Much has been written in the literature about the decline of bedside teaching in recent years, for a variety of plausible reasons, including the increasing healthcare demands, and the reliance on imaging modalities and advanced technologies to aid in complex diagnoses [5]. In 2020, an unprecedented barrier to bedside teaching emerged in the form of the Covid-19 pandemic, where almost all student-patient interactions were halted to prevent the further spread of the virus [6].

The involvement of a willing patient for bedside teaching is essential, and for this study, we propose the importance of listening to our patients’ experiences of bedside teaching, especially in the context of the Covid-19 pandemic. Whilst bedside teaching is a vital part of medical education, patients are under no obligation to participate. Previous studies have demonstrated benefits for patients participating in bedside teaching, with a key purported benefit being that patients have increased knowledge of their illness [7]. It has also been shown that the majority of patients feel that students make the hospital environment more comfortable and friendly [8].

The theoretical basis for this research centred on the sociocultural theory, which assumes that learning is a social process and happens in a social setting such as the clinical environment [9]. The purpose of this qualitative study was to gain insights into the patient’s perspective of meeting medical students in this changing clinical environment, and contributing to the education of such students, to ensure that they graduate as capable doctors.

## Methods

Twelve patients at Mayo University Hospital who partook in bedside teaching sessions were invited to take part in a semi-structured interview, regarding their experiences. Bedside tutorials took place between January and June 2022 and involved between 2 and 4 medical students. An information sheet was provided to the patients outlining the interview questions and purpose of the interview and ensuring that confidentiality was guaranteed 24 h before the interview was conducted. Ethical approval was obtained from the University of Galway College of Medicine, Nursing and Health Sciences Research Ethics Committee. The decision to carry out face-to-face interviews was considered fitting for this study, as the focus was on gaining insight and understanding, relating to the lived experience of patients who had been examined as part of a bedside tutorial. Following recruitment, informed written consent was acquired from all 12 participants, all of whom understood that they could withdraw consent at any point during or after the interview process. Each participant was additionally asked to consent to the possibility of being quoted directly but anonymously in the final write-up. The interviews were conducted by just one of the researchers, using an interview guide (Appendix), based on the one developed by Carty et al. [10], thus ensuring consistency across all of the one-to-one interviews. All interviews were audio-recorded with the permission of the participants, in order to facilitate transcription and analysis of the results. Face validity was the chosen method to ensure that the interview questionnaire used was appropriate for the purpose of the research. The interview’s semi-structured format allowed for a conversational interview with greater scope to enhance responses whilst avoiding the risk of asking leading questions. Thematic analysis of the data was initially carried out by each of the researchers independently, by becoming more familiar with the data, coding appealing facts and identifying potential themes. This permitted the researchers to collaboratively conclude the details of each theme and ensure that all related back to the objectives of the research.

## Results

Four themes emerged following a thorough analysis of the collected data as follows: the importance of the hidden curriculum, patient philanthropy, perceived personal benefit for the patient and lack of concern surrounding Covid. Tables 1, 2, 3 and 4 provide descriptive examples for each theme.

**Table 1:** Descriptive examples of Theme 1—*The importance of the hidden curriculum to the patient*

Theme	Explanation of theme and descriptive examples
The importance of the hidden curriculum to the patient	Participants recognised the effort that was being made to teach communication skills and the use of role modelling, in order to instil an empathic bedside manner when approaching patients
	(P4) “…part of the teaching was, she actually informed them how to approach, how to approach the patient. Which I, I reckon in the past, they weren’t taught things like that and so developed their own bedside manner which sometimes wasn’t great…”
	(P8) “…they talk to me you know, they tell me what they’re doing and why they’re doing it. I mean that’s wonderful for the patient……………..I liked the way they came and explained why they were here, what they were going to do”
	(P10) “Oh no she seemed to be kind of explaining things and like anything the student wanted to do to me he explained everything first and asked for permission and everything, which I thought was very good”
	(P11) “…getting that experience with patients, being very clear, being more approachable. And I think that can’t happen fast enough really”
	(P12) “…but they said they were going to do something, they brought it kind of layman’s language and told me what they were going to do next. It was comforting, it was relaxing”

**Table 2:** Descriptive examples of Theme 2—*Patient philanthropy*

Theme	Explanation of theme and descriptive examples
Patient philanthropy	Participants identified the importance of their role as patients in the education of medical students and perceived it as their duty to contribute for future safe patient care
	(P5) “Well, I don’t know if there was much benefit to me exactly but if it makes you all better doctors it’s a benefit to everyone”
	(P8) “Well yes, if it’s for their betterment then why not?”
	(P9) “For me personally, it was something I was giving sort of back because I’m receiving treatment at the moment. And it’s probably my way of giving back a bit”
	(P11) “By opening up to sort of being part of that teaching process as a patient I think it’s got to improve things for the future”
	(P7) “I do feel like there is a responsibility because we all need doctors, young people maybe not so much early on, but at some point”

**Table 3:** Descriptive examples of Theme 3—*Perceived personal benefit for the patient*

Theme	Explanation of theme and descriptive examples
Perceived personal benefit for the patient	Participants recognised the advantages of taking part in bedside teaching sessions
	(P4) “He’ll be more chatty, he’ll be more open, you might even make him feel better even though you’ve given him no medication……..as a member of the public I get to have a voice”
	(P6) “Well you would learn a little bit, just the doctors won’t do that to you they do everything very fast………and I was telling about myself, I felt a bit better in myself”
	(P7) “Breaks the monotony for me”
	(P10) “…if we can’t have student doctors to make good doctors we’ll say, well who’s going to help the likes of me or anybody else down the line, you know?”

**Table 4:** Descriptive examples of Theme 4—*Lack of concern surrounding Covid*

Theme	Explanation of theme and descriptive examples
Lack of concern surrounding Covid	Despite the upheaval during the Covid pandemic, participants were unconcerned regarding any exposure risks associated with taking part in teaching sessions
	(P1) “…it wouldn’t really bother me at the same time life must go on”
	(P3) “No because I’ve had my 4 injections”
	(P7) “No, not at all, Covid is Covid and hopefully we’re gradually putting it a little bit behind us”
	(P11) “No, no. None at all, I mean, I think all the controls that have been in since Covid and you know it’s been top notch really”
	(P12) “No, because while I am 67 years of age I have two vaccinations and two boosters so I wasn’t really concerned”

## Discussion

### The importance of the hidden curriculum to patients

“The hidden curriculum” refers to a set of implicit messages about values, norms and attitudes that learners infer from the behaviour of individual role models as well as from group dynamics, processes, rituals and structures [11]. Bedside teaching is a key tool in the delivery of these messages in medicine, to develop students’ interpersonal qualities [12]. It has been recognised that patients place equal importance on these qualities with technological competence [13], and our study has echoed this. In addition, our study shows that patients place value on positive role modelling by the medical tutor, principally with regard to informed consent, positive communication, appropriate explanation and approachability. It was interesting to see patients notice the benefit of bedside teaching for students to learn these key skills, and it appears from our study that patients found medical students to be performing well in this regard.

### Patient philanthropy

Many patients conveyed a sense of allowing the bedside tutorial to proceed as a form of giving back, recognising the importance of bedside tutorials as a tool for preparing medical students to be empathetic, competent doctors in the future, for the benefit of the wider community and not just themselves. This is echoed by Coleman et al. [14], where altruism was found to be one of the two main driving forces behind patient participation in bedside teaching, the other being personal gain. Our study highlights the awareness that patients have regarding their role in teaching medical students and confirms the positive experience it can be for them, even in coping with their illness as inpatients. This is an important realisation for clinical educators, reiterated by Rockey et al., who remind us to appreciate and acknowledge this valuable contribution, based on the patient’s desire to be helpful rather than out of obligation [15].

### Perceived personal benefit

Patients gave a range of reasons as to why they personally felt they had enjoyed and/or benefitted from the bedside tutorial itself. Many expressed that at times they felt somewhat out of the loop when it came to communication with their own physicians and that someone taking the time to sit down with them and examine them in depth, allowing them time to ask questions and let the tutor/students know if they did not understand what was going on, was refreshing and allowed them to feel more on an equal footing. Many patients simply enjoyed chatting with anyone at all, breaking up the monotony of being confined to a bed for the day, a sentiment which was also found by Coleman et al. [13], where patients stated it gave them an opportunity for companionship. Intertwined with the theme of patient philanthropy, many felt that helping these medical students to become better doctors was aiding them should they require more medical care in the future.

### Lack of concern surrounding Covid

Patients overall seemed to express little to no concern regarding Covid in relation to the bedside tutorial. Eleven out of 12 patients interviewed stated they had no issues. Many patients qualified their lack of concern, citing reasons such as the infection control measures that were in place, having received Covid vaccination and also simply that life has to go on. It is clear from the patients’ responses that despite bedside tutorials being an optional undertaking for them and not directly relevant to their treatment, they were still happy to proceed. This seems to be in direct contrast to previous studies which demonstrated high levels of fear and anxiety surrounding hospital admission and Covid transmission [16, 17]. These studies were undertaken earlier in the pandemic, as one of our patients stated, 2 years into the pandemic, “life must go on”.

## Conclusion

Medical students need to encounter patients in the clinical environment in order to become familiar with important clinical signs and symptoms that they will encounter in their careers [18]. Research has frequently focussed on the medical student or clinical teacher opinions regarding bedside teaching, without much consideration for the patients who facilitate the teaching sessions. Our study, although small, shows that patients do not consider themselves to be passive participants in teaching sessions and recognise the role that they have in contributing to the education of medical students. In addition, educators can benefit from patient feedback regarding their performance at bedside tutorials in an effort to enhance student learning for future encounters. Further research needs to be conducted, with a larger sample size, to explore this further.

## Data Availability

The data will be available upon reasonable request by contacting the corresponding author.

## Appendix. Interview guide

1. What impressions or experiences did you have of student doctors before this encounter?
2. What impressions or experiences did you have regarding how student doctors are trained today?
3. What impressions or experiences did you have of clinical teachers before this encounter?
4. What do you think of how the teacher handled the teaching session with the student doctors?
5. What impressions did you have about the communication skills and bedside manners of the student doctors and clinical teacher?
6. Were you happy with the language used, e.g. jargon used or avoided?
7. Was your privacy and confidentiality respected during the session?
8. What do you think was the benefit to you in taking part in the teaching session?
9. Would you have preferred the teaching session to have been conducted by your consultant or their team of doctors?
10. What would affect your decision to participate in bedside teaching in the future?
11. Did you have any concerns about participating in a bedside tutorial during and after the Covid pandemic?
12. Were you concerned about infection control during the session?
13. Would your willingness to participate in teaching change if the teaching was for doctors rather than medical students?
14. Were you happy with the number of student doctors who were present?
15. How can we improve the teaching experience for you or other patients?
16. Do you feel you have a place in educating student doctors?
17. If you could change anything about the teaching what would it be?
18. Is there anything else you would like to say about your experience about the teaching, e.g. why you liked it or disliked it?
